# Efficacy and safety of orelabrutinib plus R-CDOP as first-line therapy in diffuse large B-cell lymphoma with high-risk CNS-IPI: a prospective, multicenter, single-arm, phase II study

**DOI:** 10.3389/fonc.2026.1976858

**Published:** 2026-10-21

**Authors:** Yiding Guo, Jun Wang, Huyi Lei, Changqian Bao, Panpan Chen, Lihong Shou, Qunyi Guo, Beili Hu, Yongsheng Xie, Shuhan Zhong, Wenbin Qian, Xibin Xiao

**Affiliations:** 1 Department of Hematology, Zhejiang University, Hangzhou, Zhejiang, China; 2 Department of Hematology, Changde Hospital, Xiangya School of Medicine, Central South University (First People’s Hospital of Changde City), Changde, Hunan, China; 3 Department of Hematology, The Affiliated Hospital of ShaoXing University (ShaoXing Municipal Hospital), Shaoxing, Zhejiang People’s Republic of China, Shaoxing, Zhejiang, China; 4 Department of Hematology, The Second Affiated Hospital Zhejiang University School of Medicine, Hangzhou, Zhejiang, China; 5 Department of Hematology, Huzhou Central Hospital, Affiliated Central Hospital of Huzhou University, Huzhou, Zhejiang, China; 6 Department of Hematology, Taizhou Hospital of Zhejiang Province, Taizhou, Zhejiang, China; 7 Department of Hematology, The Second Affiliated Hospital of Jiaxing University, Jiaxing, Zhejiang, China

**Keywords:** CNS relapse, CNS-IPI, diffuse large B-cell lymphoma, first-line therapy, orelabrutinib

## Abstract

**Background:**

Diffuse large B-cell lymphoma (DLBCL) patients central nervous system international prognostic index (CNS-IPI) ≥4 have a high risk of central nervous system (CNS) relapse and poor prognosis. Orelabrutinib, a next-generation Bruton’s tyrosine kinase (BTK) inhibitor with excellent blood-brain barrier (BBB) penetration. This study evaluated orelabrutinib plus R-CDOP as first-line therapy in this high-risk population.

**Methods:**

This prospective, multicenter, single-arm, phase II trial (ClinicalTrials.gov: NCT06290817) enrolled untreated DLBCL patients with CNS-IPI ≥4. Patients received six 21-day cycles of orelabrutinib (150 mg orally once daily) plus R-CDOP. The primary endpoint was objective response rate (ORR) at the end of the induction; secondary endpoints included complete response (CR) rate, progression-free survival (PFS), overall survival (OS), cumulative incidence of CNS relapse, and safety.

**Results:**

Between March 2021 and September 2024, 44 patients were enrolled and constituted the intention-to-treat (ITT) population. Median age was 63 years, 64.9% had double-expressor lymphoma. In the ITT population, the ORR at the end of induction was 70.5% (31/44, 95% confidence interval [CI]: 55.3–82.1), with a CR rate of 50.0% (22/44, 95% CI: 35.1–64.9). In the per-protocol population of 37 evaluable patients, the ORR and CR rate at the end of induction were 75.7% (28/37, 95% CI: 59.9–86.9) and 59.5% (22/37, 95% CI: 43.4–73.9), respectively. At a median follow-up of 22.6 months, median PFS was not reached. The 1- and 2-year PFS rates were 77.6% (95% CI: 64.5–90.7) and 57.4% (95% CI: 40.9–73.9). Median OS was 38.1 months, with 1- and 2-year OS rates of 93.8% (95% CI: 86.2–100) and 90.2% (95% CI: 80.4–100). The 1- and 2-year cumulative incidences of CNS relapse were 0% and 9.3%, respectively. All patients had treatment-related adverse events (TRAEs); grade 3–4 TRAEs occurring in 67.6%, mainly neutropenia (59.5%), anemia (24.3%), and thrombocytopenia (16.2%). No unexpected toxicities or treatment-related deaths were observed.

**Conclusion:**

In previously untreated DLBCL patients with high-risk CNS-IPI, orelabrutinib plus R-CDOP demonstrated promising antitumor activity and a low observed rate of CNS relapse. Hematologic toxicity was frequent, but was clinically manageable with standard supportive care. These findings should be considered hypothesis-generating and warrant validation in randomized controlled trials.

**Clinical trial registration:**

ClinicalTrials.gov, identifier NCT06290817.

## Introduction

Diffuse large B-cell lymphoma (DLBCL) is the most common pathological subtype of non-Hodgkin lymphoma (NHL), accounting for 30%–40% of newly diagnosed cases (1). Although the addition of rituximab to cyclophosphamide, doxorubicin, vincristine, and prednisone (R-CHOP) has substantially improved overall outcomes, central nervous system (CNS) relapse remains a formidable clinical challenge; once CNS recurrence occurs, the median survival is less than 6 months (2). The effective prevention of CNS relapse, particularly in high-risk patients, remains one of the most critical unresolved issues in DLBCL management.

The CNS International Prognostic Index (CNS-IPI), which integrates clinical parameters, including age, lactate dehydrogenase (LDH) level, performance status, number of extranodal involvement sites, and Ann Arbor stage, has been validated as a robust tool for stratifying CNS recurrence risk (3). Patients with scores of 4–6 are classified as high-risk, with a CNS relapse risk exceeding 10%, and some ultra-high-risk subgroups may reach 15–32.5% (4). However, despite well-established risk stratification, standardized and effective first-line CNS prevention strategies for this high-risk population remain elusive. Intrathecal chemotherapy and systemic high-dose methotrexate (HD-MTX) are the most commonly used approaches; however, their prophylactic value is controversial and they carry substantial toxicity (5–7).

From a mechanistic perspective, the proliferation and survival of DLBCL are highly dependent on sustained activation of the B-cell receptor (BCR) signaling pathway, particularly in aggressive subtypes, such as non-germinal center B-cell-like (non-GCB) and double-expressor lymphoma (DEL) (8). Bruton’s tyrosine kinase (BTK) is a critical downstream signaling molecule in the BCR pathway and has emerged as an important therapeutic target (9). The first-generation BTK inhibitor ibrutinib has demonstrated clinical activity, but is limited by off-target toxicities (10). Orelabrutinib, a next-generation irreversible BTK inhibitor, exhibits substantially improved kinase selectivity and, more importantly, possesses excellent blood-brain barrier (BBB) penetration, achieving a median cerebrospinal fluid (CSF) concentration of 28.7 ng/mL and median unbound CSF-to-plasma ratio of 59.8% (11, 12). This unique pharmacokinetic property theoretically endows orelabrutinib with a dual functionality: systemic antitumor activity combined with local CNS protection. In contrast, zanubrutinib, another next-generation BTK inhibitor, has limited CSF exposure; although tirabrutinib has shown some CNS penetration, clinical data remain insufficient (13, 14). The distinctive pharmacokinetic advantage of orelabrutinib provides a robust mechanistic rationale for its clinical application in patients with DLBCL at a high risk of CNS recurrence.

Beyond targeted therapy, optimization of the chemotherapy backbone may further improve outcomes. The R-CDOP regimen, which replaces conventional doxorubicin with liposomal doxorubicin (reduced cardiotoxicity) and vincristine with vinorelbine (reduced neurotoxicity), represents a rationally modified backbone aimed at maintaining efficacy while reducing specific long-term toxicities (15–17). Based on this rationale, we designed an O-RCDOP regimen (orelbrutinib plus R-CDOP) and conducted this prospective, multicenter, single-arm phase II study to systematically evaluate its antitumor activity, CNS-preventive effect, and safety profile in DLBCL patients with high-risk CNS-IPI.

## Materials and methods

### Study design and oversight

This prospective, multicenter, single-arm, phase II trial was conducted at four centers in China. The protocol was approved by the Ethics Committee of the Second Affiliated Hospital of Zhejiang University (approval No. 2023-0465, dated April 24, 2023) and subsequently by the Institutional Review Boards of all participating centers. The study was conducted in accordance with the principles of the Declaration of Helsinki and the Good Clinical Practice guidelines. Written informed consent was obtained from all patients before any study-specific procedures. This trial was registered at ClinicalTrials.gov (identifier: NCT06290817).

### Patients

Eligibility criteria included: (1) age ≥18 years; (2) newly diagnosed, histologically confirmed CD20-positive DLBCL (including transformed DLBCL); (3) CNS-IPI score of 4–6 (indicating high risk of CNS recurrence); (4) no prior anti-lymphoma therapy (except corticosteroids for symptom control); (5) at least one measurable lesion (longest diameter >1.5 cm); (6) Eastern Cooperative Oncology Group (ECOG) performance status of 0–3; and (7) adequate organ function.

All patients underwent baseline CNS staging before enrollment. Contrast-enhanced brain MRI was performed in all patients unless contraindicated (e.g., pacemaker, severe claustrophobia), in which case contrast-enhanced brain CT was used. Lumbar puncture with CSF cytology and, when sufficient material was available, CSF flow cytometry was performed in patients without contraindications (e.g., coagulopathy, increased intracranial pressure). Ophthalmologic evaluation, including slit-lamp examination and fundoscopy, was performed in patients with visual symptoms or ocular complaints. Patients with imaging or cytological evidence of CNS involvement were excluded. Additional exclusion criteria included: (1) active hepatitis B or C virus infection; (2) known HIV infection; (3) history of other active malignancies within the past 5 years; (4) severe cardiovascular disease (including uncontrolled arrhythmia, New York Heart Association [NYHA] class III or IV congestive heart failure, or myocardial infarction within 6 months); and (5) pregnancy or lactation.

### Treatment

Eligible patients received the O-RCDOP induction regimen for six 21-day cycles. The regimen consisted of oral orelabrutinib (150 mg once daily on days 1–21), rituximab (375 mg/m^2^ intravenously on day 0), cyclophosphamide (750 mg/m^2^ intravenously on day 1), liposomal doxorubicin (30 mg/m^2^ intravenously on day 1), vinorelbine (25 mg/m² intravenously on day 1; maximum single dose, 40 mg), and prednisone (100 mg orally on days 1–5).

To enhance safety in elderly patients, pre-specified age-stratified dose reductions were applied; for patients aged 70–80 years, cyclophosphamide and liposomal doxorubicin were reduced to 70–80% of the standard dose, and for those over 80 years, to 50–60% of the standard dose. Prophylactic granulocyte colony-stimulating factor (G-CSF) was administered according to institutional guidelines. Patients who achieved a deep response and were deemed eligible underwent autologous hematopoietic stem cell collection after 3–4 cycles for potential consolidation with autologous stem cell transplantation (ASCT) after the completion of induction therapy.

### Assessments and endpoints

Tumor response was assessed using contrast-enhanced computed tomography (CT) or positron emission tomography-CT at baseline, after cycles 2 and 4, and at the end of induction therapy (cycle 6). The responses were classified according to Lugano 2014 criteria (18). The primary endpoint was the objective response rate (ORR) at the end of six cycles of induction therapy, defined as the proportion of patients achieving complete response (CR) or partial response (PR). Secondary endpoints included: (1) CR rate; (2) progression-free survival (PFS), defined as the time from treatment initiation to disease progression, relapse, or death from any cause; (3) overall survival (OS), defined as the time from treatment initiation to death from any cause; (4) cumulative incidence of CNS relapse, defined as the first occurrence of lymphoma in the brain parenchyma, leptomeninges, spinal cord, or eyes confirmed by imaging or cytology; and (5) safety. Adverse events (AEs) were recorded and graded according to the National Cancer Institute Common Terminology Criteria for Adverse Events, version 5.0 (NCI-CTCAE v5.0) (19).

### Sample size calculation

The initial sample size calculation was based on the originally specified primary endpoint of the CNS recurrence rate. Assuming a 2-year CNS recurrence rate of 10% in a historical high-risk cohort receiving R-CHOP-based therapy and that the O-RCDOP regimen could reduce this rate to 2% with a one-sided alpha of 0.05, power of 80%, and dropout rate of 5%, 62 patients were initially planned for enrollment. However, because CNS relapse events are infrequent within a short follow-up period and ORR is a more immediate indicator of antitumor activity, the protocol was amended to change the primary endpoint to ORR. The amendment was approved by the Ethics Committee of the Second Affiliated Hospital of Zhejiang University on May 8, 2023. At the time of the amendment, 18 patients had been enrolled, and no patients had completed the primary endpoint assessment; therefore, the amendment did not affect the efficacy evaluation of any patient. The revised sample size calculation assumes a historical ORR of 60% with standard therapy and an anticipated improvement to 80% with O-RCDOP. Using a one-sided alpha of 0.05, 80% power, and a 5% dropout rate, 35 evaluable patients were required.

### Statistical analysis

The primary efficacy analysis was based on the ITT population. For the ITT analysis, patients who withdrew early because of progressive disease (PD) or stable disease (SD) were counted as non-responders for the primary endpoint analysis, rather than carrying forward their last response. The PP analysis was performed as a sensitivity analysis in patients who completed at least one post-baseline efficacy assessment. For patients lost to follow-up (none in this study), the data were censored at the last known follow-up time point.

Continuous variables are described using medians and ranges, and categorical variables are summarized as frequencies and percentages. ORR and CR rates are presented with their corresponding 95% confidence intervals (CIs), calculated using the Clopper-Pearson exact method. For comparisons of ORR/CR rates across multiple time points (cycles 2, 4, and 6), the Bonferroni method was applied for multiple comparison correction, with a corrected significance threshold of α’ = 0.017. Time-to-event endpoints (PFS and OS) were estimated using the Kaplan-Meier method, with median PFS and OS reported with corresponding 95% CIs. The cumulative incidence of CNS relapse was analyzed using a competing risk model, with death without CNS relapse and non-relapse mortality considered as competing events. All statistical analyses were conducted using the SAS software (version 9.4; SAS Institute, Cary, NC, USA).

## Results

### Baseline patient characteristics

A total of 44 patients were enrolled between March 2021 and September 2024. Of these, 37 completed at least one post-baseline efficacy assessment and constituted the PP population, whereas all 44 were included in the ITT analysis set. The 7 patients excluded from the PP population had numerically poorer baseline features than the 37 evaluable patients. None of the 7 excluded patients had a CNS-IPI score of 6 (0/7, 0% vs. 6/37, 16.2%). However, the excluded patients had a higher proportion of poor ECOG performance status: 2 patients had ECOG PS 3, 1 had ECOG PS 4, and 1 had ECOG PS 5, compared with 8/37 (21.6%) with ECOG PS 3 in the evaluable population. In addition, double-expressor lymphoma was more frequent among excluded patients (6/7, 85.7% vs. 24/37, 64.9%). Five of these 7 patients withdrew because of early progressive disease, one because of stable disease, and one because of insufficient treatment cycles and missing key efficacy data. These findings suggest that the PP analysis may overestimate treatment efficacy relative to the ITT analysis. (**Figure 1**).

**Figure 1:**
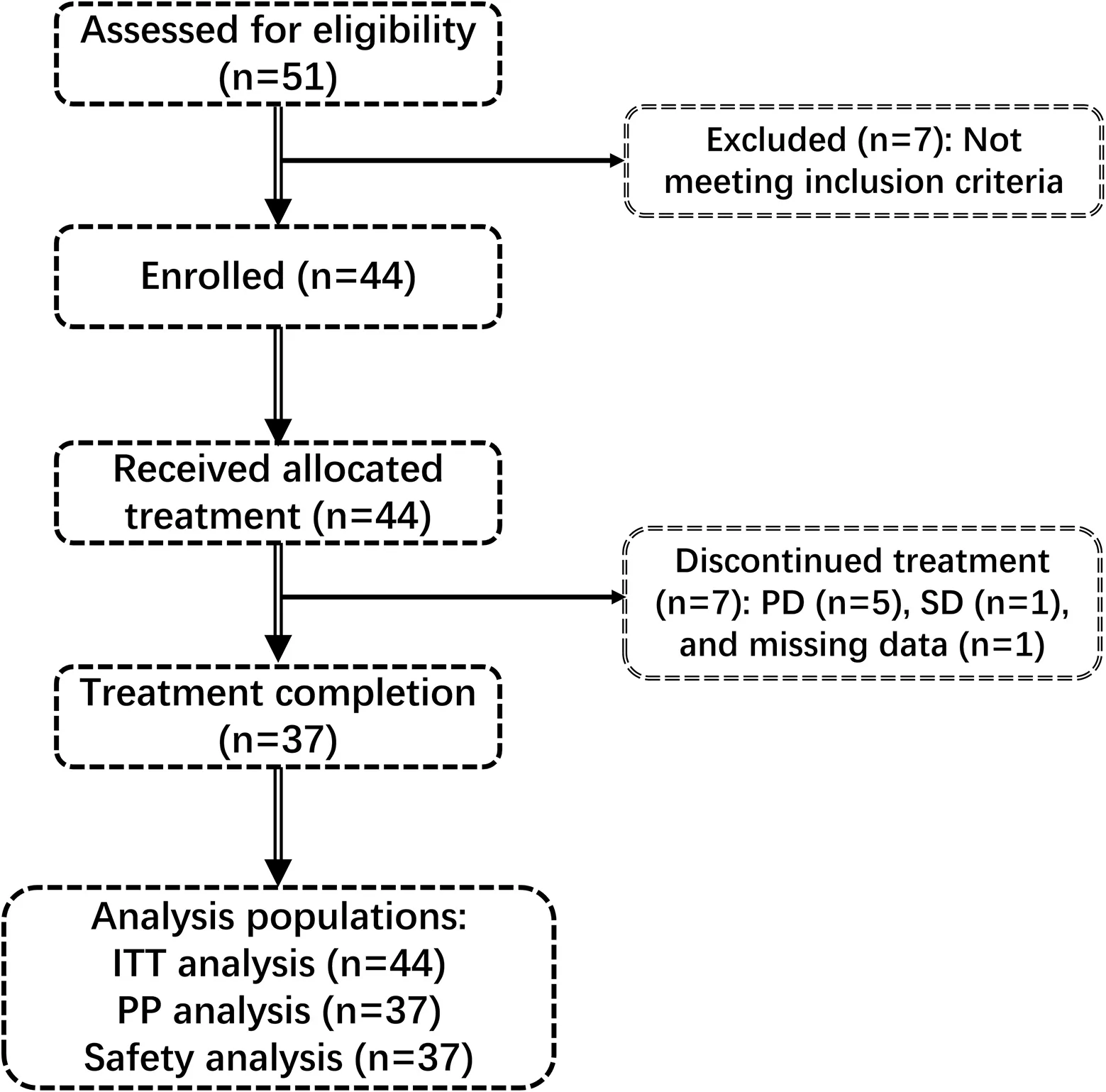
CONSORT diagram showing patient enrollment, treatment completion, and analysis populations.

Among the 37 evaluable patients, contrast-enhanced brain MRI was performed in 34 patients (91.9%), and contrast-enhanced brain CT in 3 patients (8.1%) due to contraindications to MRI. Lumbar puncture was performed in 29 patients (78.4%); CSF cytology was negative in all evaluated patients, and CSF flow cytometry was available in 18 patients (48.6%), all of which showed no evidence of CNS involvement. Ophthalmologic evaluation was performed in 6 patients (16.2%) with ocular symptoms, and none showed lymphomatous involvement.

The baseline demographic and clinical characteristics are summarized in **Table 1**. The median age was 63 years (range, 41–89 years), and 13 patients (35.1%) were aged > 70 years old. Most patients were male (24 patients, 64.9%) and had an ECOG performance status of 0–2 (29 patients, 78.4%). Immunohistochemical analysis revealed that 24 patients (64.9%) had double-expressing lymphoma (co-expression of MYC and BCL-2). All patients had a CNS-IPI score of 4–6, confirming their high-risk status for CNS relapse; 15 patients (40.5%) scored 4, 16 (43.2%) scored 5, and 6 (16.2%) scored 6. High-risk extranodal involvement (testis, breast, adrenal gland, kidney, uterus/ovary, or epidural space) was observed in 9 patients (24.3%), confirming that the cohort included a substantial proportion of patients with recognized high-risk features for CNS relapse.

**Table 1:** Baseline demographic and clinical characteristics of patients with high-risk CNS-IPI diffuse large B-cell lymphoma treated with orelabrutinib plus R-CDOP (N = 37).

Characteristic	No.%
Median age, years (range)	63 (41–89)
Age >70 years, n (%)	13 (35.1)
Sex, n (%)	
Male	24 (64.9)
Female	13 (35.1)
ECOG PS, n (%)	
0-1	22 (59.5)
2	7 (18.9)
3	8 (21.6)
CNS-IPI Score, n (%)	
4	15 (40.5)
5	16 (43.2)
6	6 (16.2)
Double-Expressor (MYC & BCL-2), n (%)	24 (64.9)
Cell-of-Origin (by IHC), n (%)	
GCB	11 (29.7)
Non-GCB	26 (70.3)
High-risk extranodal sites, n (%)	9 (24.3)
Testis	2 (5.4)
Breast	2 (5.4)
Adrenal gland	2 (5.4)
2 (5.4)
Epidural	1 (2.7)

CNS-IPI, central nervous system international prognostic index; ECOG PS, Eastern Cooperative Oncology Group performance status; GCB, germinal center B-cell like; IHC, immunohistochemistry.

### Efficacy

All 44 enrolled patients were included in the ITT analysis, patients who withdrew early because of PD or SD were counted as non-responders. At the end of cycle 6, the ITT ORR was 70.5% (31/44, 95% CI: 55.3–82.1), with a CR rate of 50.0% (22/44, 95% CI: 35.1–64.9). In the PP population of 37 evaluable patients, the ORR was 75.7% (28/37, 95% CI: 59.9–86.9), with a CR rate of 59.5% (22/37, 95% CI: 43.4–73.9). The details regarding ORR after 2, 4, and 6 cycles of O-RCDOP induction therapy is shown in **Figure 2**. After Bonferroni correction for multiple comparisons, the CR rate in cycle 6 was significantly higher than that in cycle 2 (*P* < 0.001). Seven patients who achieved CR underwent ASCT as consolidation therapy.

**Figure 2:**
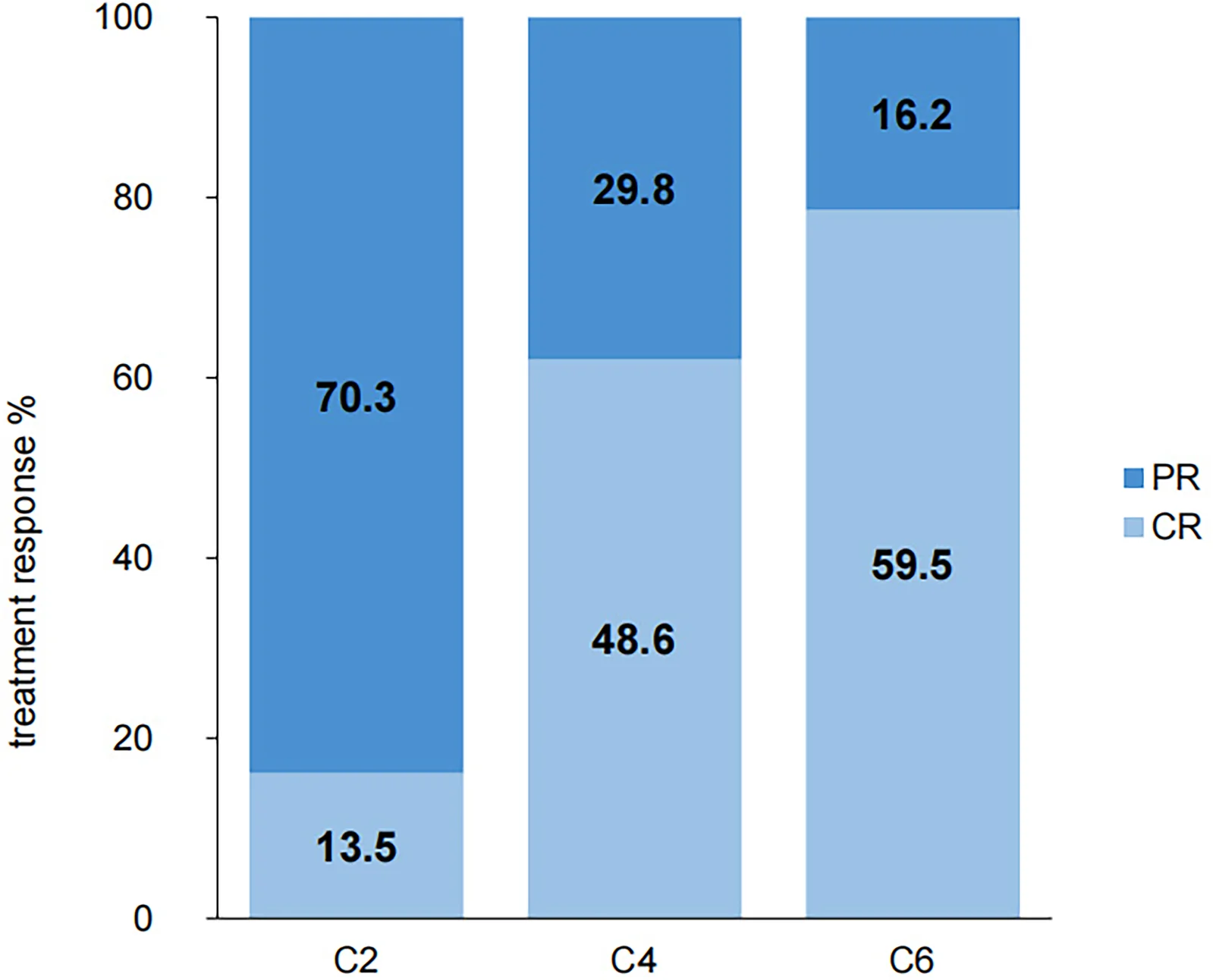
Best overall response rates after 2, 4, and 6 cycles of O-RCDOP induction therapy.

### Survival outcomes and CNS relapse

With a median follow-up of 22.6 months (range, 1.6–50.9), the median PFS was not reached [NR (95%CI: 22.1–NE)] (**Figure 3**). The estimated 1- and 2-year PFS rates were 77.6% (95%CI: 64.5–90.7) and 57.4% (95% CI: 40.9–73.9), respectively. A total of 16 PFS events occurred during follow-up. Of these, 2 were isolated CNS relapses, 12 were systemic progressions, and 2 were combined CNS and systemic progressions. The 2 CNS relapses occurred at 8.2 and 19.4 months after treatment initiation; both involved the brain parenchyma, and one also had leptomeningeal involvement. Among the 16 patients with PFS events, 11 received subsequent salvage therapy, including CAR-T cell therapy (n=4), lenalidomide-based regimens (n=3), platinum-based salvage chemotherapy (n=2), radiotherapy (n=1), and other systemic therapy (n=1). The remaining 5 patients received best supportive care or were lost to subsequent follow-up. Of the 7 patients who underwent ASCT, all were in CR at the time of transplantation and received ASCT as consolidation therapy rather than salvage therapy.

**Figure 3:**
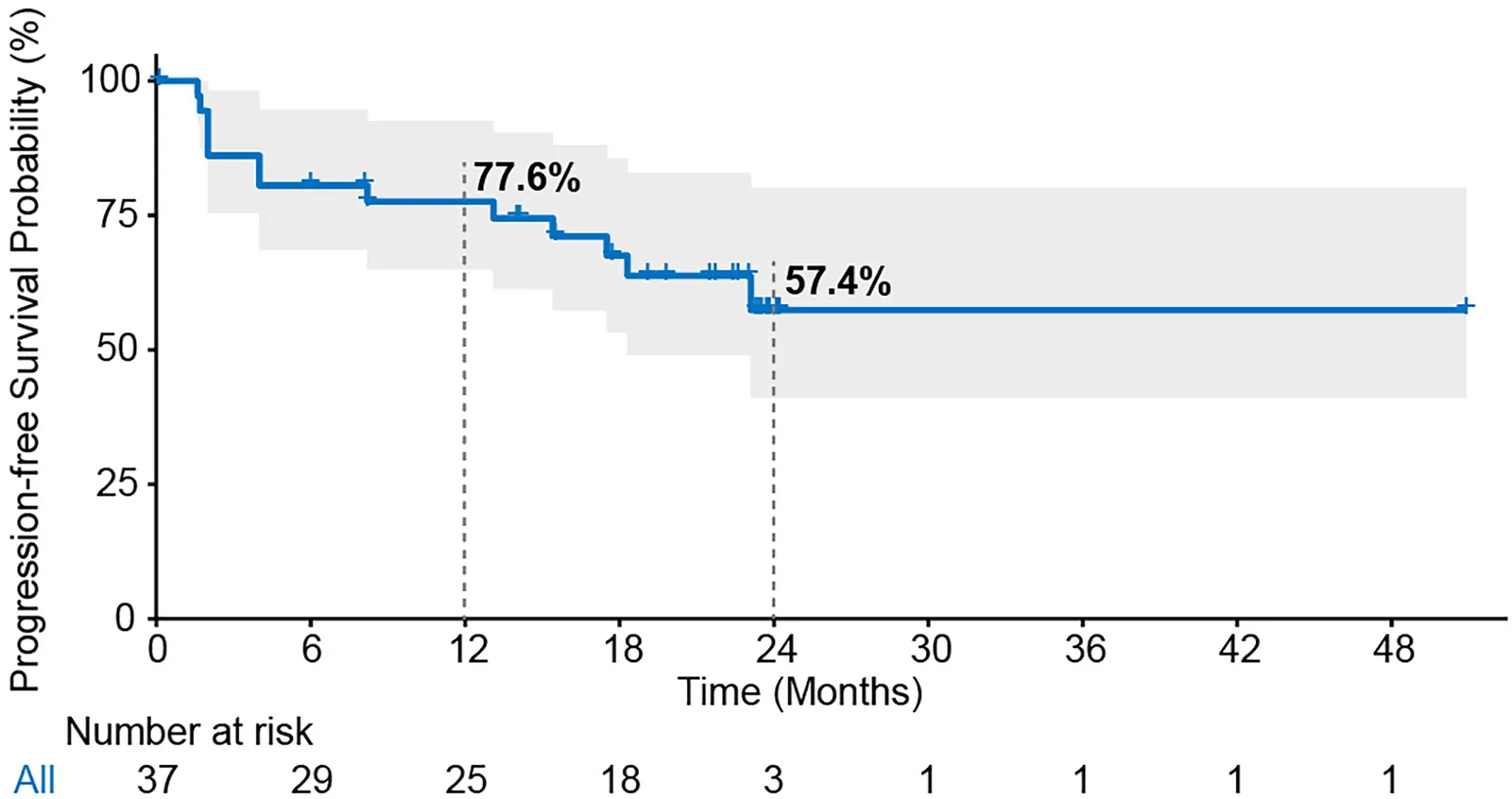
Kaplan-Meier curve of progression-free survival (PFS). PFS was defined as the time from treatment initiation to disease progression, relapse, or death from any cause.

Median OS was 38.1 months (95%CI: 30.1–NE) (**Figure 4**). Estimated 1- and 2-year OS rates were 93.8% (95%CI: 86.2–100) and 90.2% (95%CI: 80.4–100), respectively. Notably, the 2-year PFS rate (57.4%) was lower than the 2-year OS rate (90.2%), suggesting that some patients who experienced relapse or progression were still effectively salvaged by subsequent therapies, including ASCT and chimeric antigen receptor T-cell (CAR-T) therapy.

**Figure 4:**
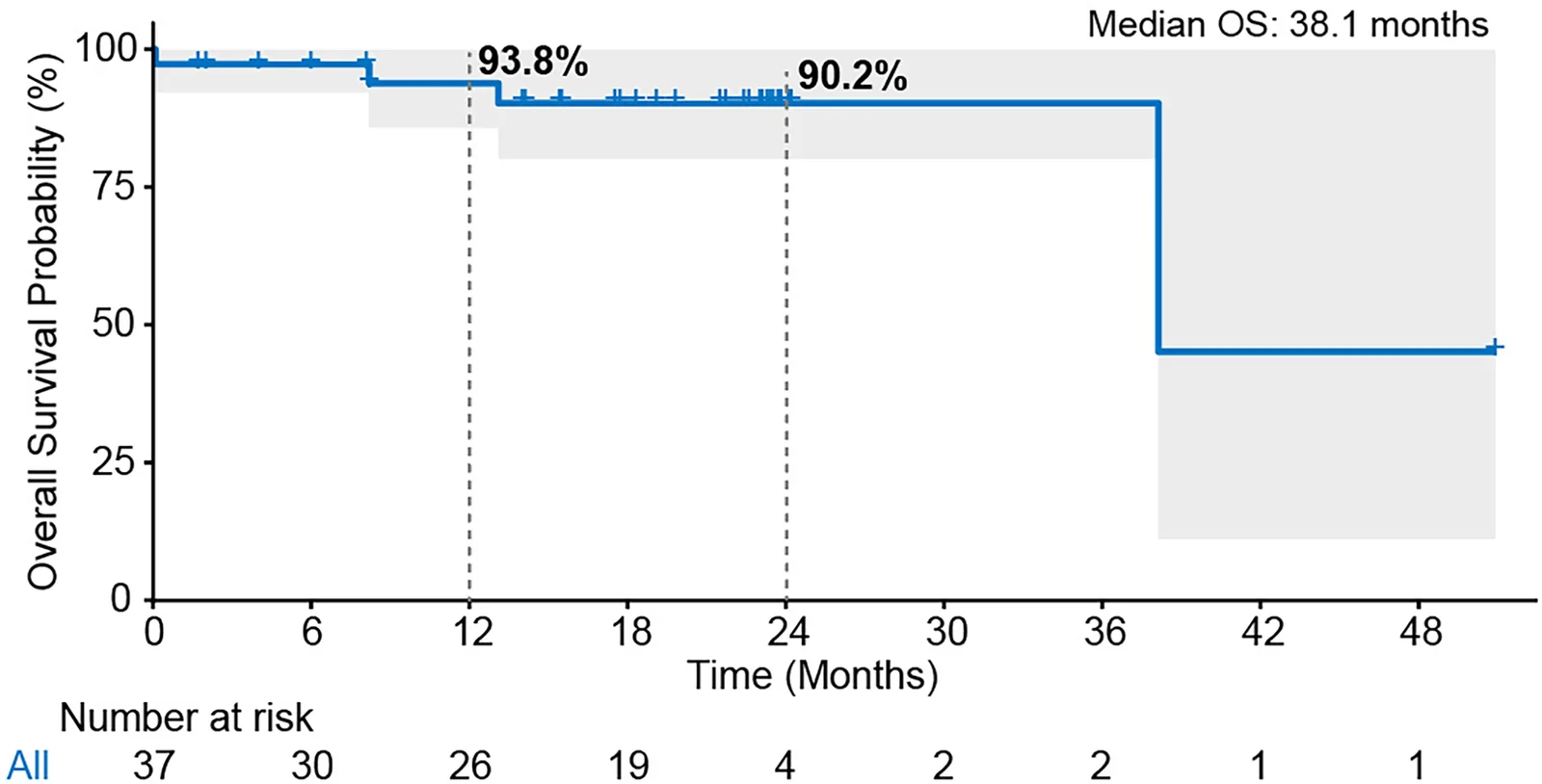
Kaplan-Meier curve of overall survival (OS). OS was defined as the time from treatment initiation to death from any cause.

The median follow-up period for this specific analysis was 19.1 months. CNS relapse occurred in two of the 37 patients (5.4%). The cumulative incidence of CNS relapse was 0% (95% CI: 0–0) at 1 year and 9.3% (95%CI: 0–21.5) at 2 years (**Figure 5**). It should be noted that with only two CNS relapse events, the stability of this estimate was limited, as reflected by the wide 95%CI upper bound, warranting cautious interpretation.

**Figure 5:**
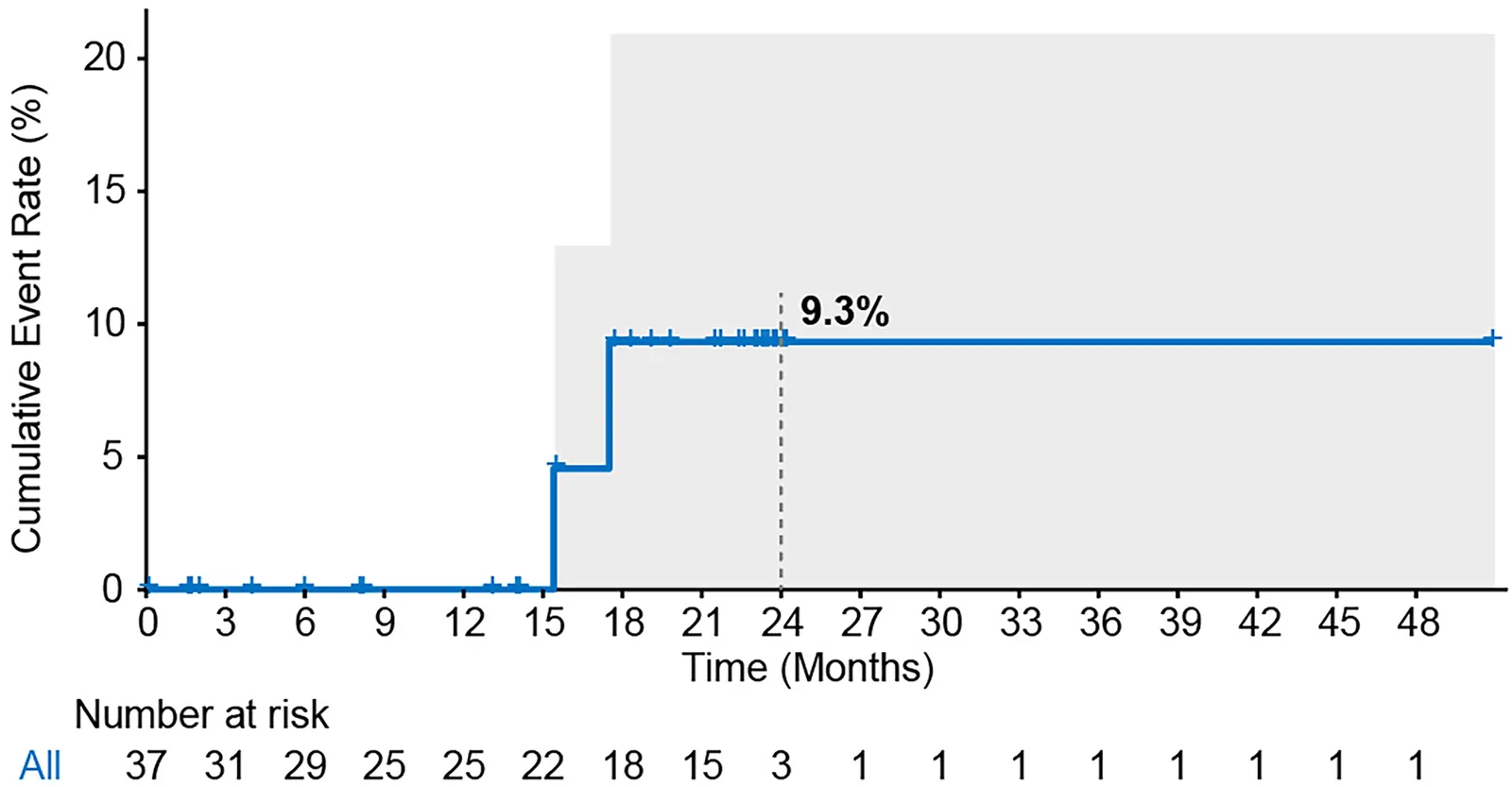
Cumulative incidence curve of central nervous system (CNS) relapse. CNS relapse was defined as the first occurrence of lymphoma involving the brain parenchyma, leptomeninges, spinal cord, or eyes, confirmed by imaging or cytology.

### Safety

The safety in all 37 patients was assessed (**Table 2**). All patients (100%) experienced at least one treatment-related adverse event (TRAE) of any grade. The most common hematological TRAEs of any grade were neutropenia (100%), anemia (91.9%), and thrombocytopenia (75.7%). Common non-hematological TRAEs included nausea (64.9%), fatigue (59.5%), and elevated transaminase levels (43.2%).

**Table 2:** Treatment-related adverse events in patients receiving orelabrutinib plus R-CDOP (N = 37).

Adverse event	All grades, n (%)	Grade 3-4, n (%)
Hematological		
Neutropenia	37 (100)	22 (59.5)
Anemia	34 (91.9)	9 (24.3)
Thrombocytopenia	28 (75.7)	6 (16.2)
Non-Hematological		
Nausea	24 (64.9)	0
Fatigue	22 (59.5)	0
Increased ALT/AST	16 (43.2)	0
Constipation	15 (40.5)	0
Diarrhea	12 (32.4)	1 (2.7)
Decreased Appetite	11 (29.7)	0
Peripheral Edema	8 (21.6)	0
Rash	6 (16.2)	0

Adverse events are graded according to the National Cancer Institute Common Terminology Criteria for Adverse Events, version 5.0. Only events occurring in ≥10% of patients in any grade are listed.

ALT, alanine aminotransferase; AST, aspartate aminotransferase.

Grade 3 or 4 TRAEs occurred in 25 (67.6%) patients. The most frequent grade 3–4 events were hematological: neutropenia (22 patients, 59.5%), anemia (nine patients, 24.3%), and thrombocytopenia (six patients, 16.2%). These rates are substantial and reflect the myelosuppressive intensity of the R-CDOP backbone in this elderly, high-risk population. All grade 3–4 hematologic events were managed with standard supportive measures, including G-CSF prophylaxis, therapeutic G-CSF, and blood component transfusion, and no patient permanently discontinued treatment or died because of toxicity. No grade 5 (fatal) TRAEs were reported. Importantly, no atrial fibrillation/flutter events or clinical tumor lysis syndromes were observed. One patient experienced Grade 3 diarrhea, which resolved with supportive care. Dose reductions or delays due to AEs were managed as per protocol, and no patient permanently discontinued treatment owing to toxicity.

## Discussion

This prospective multicenter phase II study explored the O-RCDOP regimen in patients with previously untreated DLBCL with high-risk CNS-IPI scores. In the ITT population, the ORR at the end of induction was 70.5%, and the 2-year cumulative incidence of CNS relapse was 9.3% (95% CI: 0–21.5), based on only two CNS relapse events. Because of the single-arm design, small sample size, and limited number of CNS events, these findings should be regarded as preliminary and hypothesis-generating rather than as evidence that O-RCDOP is an effective CNS-prophylactic strategy. Accordingly, the results should be interpreted as supporting the feasibility and rationale for further evaluation of a methotrexate-free approach, rather than as demonstrating a paradigm shift in CNS prevention. These results have three potential clinical implications that warrant further investigation.

This study provides a novel HD-MTX-free strategy for CNS prophylaxis in high-risk DLBCL patients. Although HD-MTX is the most commonly used CNS prophylaxis, its clinical benefits remain controversial. Recent large-scale retrospective analyses, including an international multicenter study of 1,923 patients, have consistently demonstrated that HD-MTX does not significantly reduce CNS relapse rates in high-risk patients while introducing substantial toxicity and treatment burden (6). In the present study, without HD-MTX use, the observed 2-year cumulative CNS relapse rate of 9.3% is descriptively lower than the expected rate in historical high-risk cohorts (3), the small sample size and the limited number of CNS relapse events preclude any definitive conclusion that orelabrutinib prevents CNS relapse. This study should therefore be regarded as providing preliminary evidence to support further investigation of a methotrexate-free CNS prophylaxis strategy, rather than demonstrating its efficacy. Nevertheless, this study provides the first prospective evidence for “methotrexate-free” CNS prophylaxis in a strictly defined high-risk CNS-IPI population, representing a potential paradigm shift in CNS prevention strategies.

Moreover, the O-RCDOP regimen demonstrated robust systemic antitumor activity. In this poor prognosis population, with a 64.9% prevalence of double-expressor lymphoma, the CR rate at the end of induction reached 59.5%, with a median OS of 38.1 months and a 2-year OS rate of 90.2%. These outcomes compare favorably with the historical expectations of the high-risk cohort. Compared with the Smart Start study (rituximab, lenalidomide, and ibrutinib followed by chemotherapy in newly diagnosed non-GCB DLBCL) reported by Westin et al. (20), the 2-year OS rate in our study (90.2%) was comparable to the 96.6% reported in Smart Start, but the 2-year PFS rate differed substantially (57.4% vs. 91.3%). This discrepancy is primarily attributable to fundamental differences in patient populations: Smart Start enrolled patients based on non-GCB subtype (which may include low-to-intermediate CNS-IPI cases), whereas our study mandated CNS-IPI ≥4 as a rigid entry criterion, representing a population with substantially poorer baseline prognosis. Furthermore, the progressive increase in CR rates from cycle 2 to cycle 6 (13.5% → 48.6% → 59.5%) suggests that the O-RCDOP regimen may require adequate exposure duration to achieve maximal efficacy. However, this study did not include correlative biological analyses, and the potential contribution of sustained BCR pathway inhibition to tumor microenvironment remodeling remains speculative and should be investigated in future studies.

The marked divergence between PFS and OS should be interpreted cautiously. The 2-year PFS rate (57.4%) was notably lower than the 2-year OS rate (90.2%), suggesting that some patients who experienced relapse or progression may have been effectively salvaged by subsequent therapies, including ASCT and CAR-T cell therapy. However, because post-progression treatment was not prospectively controlled and was collected retrospectively, the excellent OS cannot be attributed solely to the investigational first-line regimen, and the contribution of subsequent therapies cannot be fully separated from the effect of O-RCDOP. This pattern suggests that the O-RCDOP regimen, as a first-line therapy, not only possesses intrinsic antitumor activity but may also preserve patients’ good performance status and effectively control CNS relapse, thereby creating favorable conditions for subsequent salvage therapies. Future studies should systematically collect postrelapse treatment details to comprehensively evaluate the optimal positioning of O-RCDOPs within the DLBCL treatment pathway.

The O-RCDOP regimen demonstrated a safety profile that was clinically manageable but characterized by frequent and substantial myelosuppression. Grade 3–4 TRAEs occurred in 67.6% of patients, predominantly neutropenia (59.5%), anemia (24.3%), and thrombocytopenia (16.2%). These rates are not negligible and are at least comparable to, if not higher than, those reported with standard R-CHOP in similar populations. Importantly, all grade 3–4 hematologic events were effectively managed with standard supportive measures, including G-CSF support and blood component transfusions, and no patient permanently discontinued treatment or died because of toxicity. Importantly, we did not observe off-target toxicities commonly associated with the first-generation BTK inhibitor ibrutinib, such as atrial fibrillation, major bleeding, or invasive fungal infections. To facilitate clinical implementation of this regimen, based on our practical experience, we propose the following management recommendations: (1) routine prophylactic G-CSF after the first cycle for all patients; (2) therapeutic G-CSF for patients with neutropenia until ANC recovery to ≥2.0 × 10^9^/L; and (3) consideration of orelabrutinib dose reduction to 100 mg/d or further chemotherapy dose reduction for patients with recurrent severe neutropenia.

Importantly, we did not observe off-target toxicities commonly associated with the first-generation BTK inhibitor, ibrutinib, such as atrial fibrillation, major bleeding, or invasive fungal infections (10). This finding is consistent with the superior kinase selectivity profile (21), supporting its potential for better tolerability in elderly patients and those with comorbidities. Concurrently, the substitution of conventional doxorubicin with liposomal doxorubicin likely contributed to the absence of significant clinical cardiotoxicity in this study, which is an important consideration for patients pursuing long-term survival (22).

The CNS penetration profile of orelabrutinib was the core pharmacological rationale of this regimen design. The theoretical hypothesis underlying this study—that a highly CNS-penetrant BTK inhibitor could achieve the dual functionality of systemic antitumor activity and local CNS protection—has been supported to a certain extent by clinical data. Pharmacokinetic studies have shown that orelabrutinib achieves a median CSF concentration of 28.7 ng/mL and a median unbound CSF-to-plasma concentration ratio of 59.8%, making it one of the most potent next-generation CNS-penetrant BTK inhibitors currently known (23). Compared with other next-generation BTK inhibitors, zanubrutinib has limited CSF exposure, and while tirabrutinib has shown some CNS penetration, the clinical evidence remains insufficient (13, 14). Admittedly, the present study did not directly measure orelabrutinib concentrations in patients’ CSF, and actual CNS exposure levels require further investigation for confirmation.

This study has several limitations that should be considered when interpreting the results. First, the single-arm design is an inherent limitation of this phase II exploratory study. The absence of a concurrent randomized control group means that comparisons with historical data may be subject to selection bias, stage migration, and evolving supportive care standards. Therefore, the observed efficacy and CNS relapse rate should be regarded as hypothesis-generating rather than definitive evidence of benefit over standard R-CHOP/R-CDOP. Second, although the sample size met the prespecified statistical requirements, the limited scale of 37 evaluable patients restricts the reliability of subgroup analyses, and the observation of only two CNS relapse events results in limited estimation precision. Third, 7 patients were excluded from the PP population, including 5 with early progressive disease; these patients had numerically worse baseline prognostic features, and the PP results should therefore be interpreted with caution. Fourth, the patient population was exclusively Chinese, and given potential ethnic differences in pharmacogenomics, DLBCL molecular subtype distribution, and medical practice patterns, the generalizability of the findings to other populations warrants caution. In addition, patients with a CNS-IPI score of 6 accounted for only 16.2% (6/37) of the cohort, limiting the generalizability of the results to ultra-high-risk populations. Moreover, the protocol allowed age-stratified dose reductions in elderly patients, which introduces heterogeneity in treatment intensity and may affect the interpretation of efficacy and safety. Fifth, the patient population was exclusively Chinese, and given the potential ethnic differences in pharmacogenomics, DLBCL molecular subtype distribution, and medical practice patterns, the generalizability of the findings to other populations warrants caution. Sixth, although baseline CNS staging was systematically performed, lumbar puncture and CSF flow cytometry were not completed in all patients because of patient refusal or clinical contraindications; therefore, the possibility of occult CNS involvement at baseline cannot be entirely excluded, and the 2-year CNS relapse rate may have been underestimated. Finally, post-progression treatment data were collected retrospectively and were incomplete for some patients; therefore, the contribution of salvage therapies to the observed OS benefit cannot be fully quantified.

## Conclusion

In conclusion, in this hypothesis-generating phase II study, orelabrutinib plus R-CDOP demonstrated promising antitumor activity and a low observed rate of CNS relapse. The safety profile was clinically manageable with intensive supportive care, although grade 3–4 myelosuppression was common, which should be carefully considered in clinical practice. These findings should be considered hypothesis-generating and warrant further validation in randomized controlled trials.

## Data Availability

The raw data supporting the conclusions of this article will be made available by the authors, without undue reservation.
